# The Role of the Cerebellum in Social and Non-Social Action Sequences: A Preliminary LF-rTMS Study

**DOI:** 10.3389/fnhum.2021.593821

**Published:** 2021-02-25

**Authors:** Elien Heleven, Kim van Dun, Sara De Witte, Chris Baeken, Frank Van Overwalle

**Affiliations:** ^1^Department of Psychology and Center for Neuroscience, Vrije Universiteit Brussel, Brussels, Belgium; ^2^Biomedical Research Institute, Hasselt University, Hasselt, Belgium; ^3^Department of Head and Skin, Ghent University, Ghent, Belgium; ^4^Department of Psychiatry, University Hospital Brussels (UZ Brussel), Brussels, Belgium; ^5^Ghent Experimental Psychiatry (GHEP) Lab, Ghent University, Ghent, Belgium; ^6^Department of Electrical Engineering, Eindhoven University of Technology, Eindhoven, Netherlands

**Keywords:** cerebellum, social action sequences, mentalizing, TMS, Verbal sequencing task, Picture sequencing task

## Abstract

An increasing number of studies demonstrated the involvement of the cerebellum in (social) sequence processing. The current preliminary study is the first to investigate the causal involvement of the cerebellum in sequence generation, using low-frequency repetitive transcranial magnetic stimulation (LF-rTMS). By targeting the posterior cerebellum, we hypothesized that the induced neuro-excitability modulation would lead to altered performance on a Picture and Story sequencing task, which involve the generation of the correct chronological order of various social and non-social stories depicted in cartoons or sentences. Our results indicate that participants receiving LF-rTMS over the cerebellum, as compared to sham participants, showed a stronger learning effect from pre to post stimulation for both tasks and for all types of sequences (i.e. mechanical, social scripts, false belief, true belief). No differences between sequence types were observed. Our results suggest a positive effect of LF-rTMS on sequence generation. We conclude that the cerebellum is causally involved in the generation of sequences of social and nonsocial events. Our discussion focuses on recommendations for future studies.

## Introduction

Over the last 5 years, neuroscientists have demonstrated the robust involvement of the cerebellum in social cognition (Van Overwalle et al., 2014), although the cerebellum has been traditionally seen as a major site of non-cognitive motor and movement functioning. Specifically, the posterior part of the cerebellum (i.e., Crus 1 and 2) seems to be critical for social functioning, and its functional connectivity with cortical regions known to be involved in social processing has now been firmly established (Buckner et al., 2011; Van Overwalle et al., 2015, 2019c, 2020). In addition, clinical studies revealed social impairments of cerebellar patients compared to healthy controls (Sokolovsky et al., 2010; Hoche et al., 2016), and also revealed the impact of deficiencies in the cerebellum on social functioning in various other neuropsychiatric and neurodevelopmental disorders, such as autistic spectrum disorders, attentional deficit and hyperkinetic disorder, depression, and schizophrenia (Bauman and Kemper, 2005; Penn, 2006; Wang et al., 2014; D’Mello et al., 2015). In sum, these studies demonstrate the involvement of the cerebellum in social information processing and its connectivity to social regions and functionality in the cerebrum.

To explain the role of the cerebellum in cognition, Leggio and Molinari (2015) put forward the hypothesis that the cerebellum supports the detection and construction of internal models of sequences involving not only motor, but also purely mental elements, based on frequently processed temporally or spatially structured sequences of events. To further elucidate the social function of the cerebellum, Van Overwalle et al. (2019b) hypothesized that the cerebellum builds such internal models also of social action sequences to predict how other people’s actions will be executed. This mechanism likely allows to better anticipate action sequences during social interactions in an automatic and intuitive way and to fine-tune these anticipations, making it easier to understand social behaviors and to detect violations. The involvement of the cerebellum in social sequences is typically investigated using tasks consisting of elements of social actions that have to be put into the correct chronological order (e.g., Leggio et al., 2008; Cattaneo et al., 2012).

**FIGURE 1:**
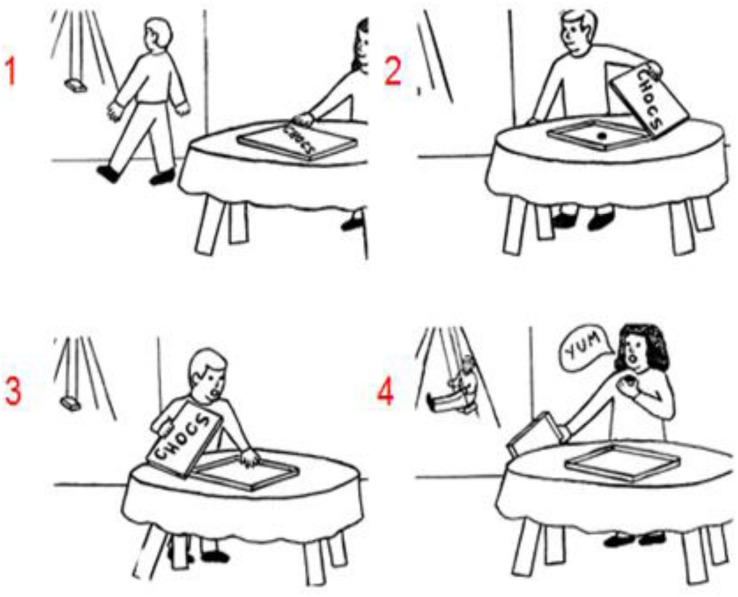
An example of a false belief sequence in the Picture Sequencing task (Langdon and Coltheart, 1999; the correct order is 2 – 1 – 4 – 3; the numbers are not shown to the participants but given here for display purposes). As in Heleven et al. (2019) participants had to select the first picture of the sequence on the screen, then the second picture, and so on. Each time, the pictures moved in the order indicated by the participant. At the end of each trial, participants could cancel and redo the trial to correct possible mistakes, or end the trial.

Recent sequencing studies investigated a key element of social understanding, which is the capacity of *mind reading* or *mentalizing* (Heleven et al., 2019; Van Overwalle et al., 2019a). This is the ability to infer and understand other peoples’ mind such as their beliefs, knowledge, or preferences that drive their actions (for a review see Van Overwalle, 2009; Schurz et al., 2014; Molenberghs et al., 2016). A key task of social mentalizing requires the understanding of *false beliefs* (for an example see Figure 1; Langdon and Coltheart, 1999), that is, the notion that other people might have a different interpretation and representation of reality because they do not know that objects were changed or moved in their absence (e.g., Baron-Cohen et al., 1985). In contrast, *true beliefs* refer to other people’s mental understanding and representation of reality as it is currently in place (and perceived by the self). To test the role of social mentalizing in the cerebellum, Van Overwalle et al. (2019a) requested participants to order pictorial sequences involving such true and false belief stories in a Picture sequencing task. The results revealed that cerebellar patients performed significantly worse than healthy matched controls when ordering false belief story sequences, but similar on routine social scripts and non-social mechanical movement sequences. In a functional magnetic resonance study (fMRI) on healthy participants, Heleven et al. (2019) observed more activation in the posterior cerebellum (i.e., Crus 1 and 2) during sequencing of false and true beliefs compared to mechanical events in a Picture and Story sequencing task (presented in cartoons and sentences, respectively). No differences were found between true and false belief sequencing. Taken together, these studies suggest that the posterior cerebellum is important for social action generation and is specifically involved in sequencing true and false beliefs.

However, these initial studies on the sequential social role of the cerebellum were limited in that they could not demonstrate that the cerebellum is necessary for social sequencing. It could be that cerebellar activation in these previous studies demonstrated merely a side or aftereffect, instead of a critical causal role. Therefore, the current study investigated the causal role of the cerebellum for social sequence generation. To do so, we stimulated the cerebellum in a non-invasive manner by applying transcranial magnetic stimulation (TMS). TMS is a useful tool to modulate the neural excitability of a targeted brain region by means of an electric field induced by a magnetic pulse using a magnetic coil (for a review on TMS on the cerebellum, see van Dun et al., 2017). Specifically, we applied low frequency repetitive TMS (LF-rTMS). Although it is generally assumed that the net effect of LF-rTMS is inhibitory, the specific effects of this technique targeting the cerebellum are not yet well-understood (van Dun et al., 2017), because of the many inhibitory projections within cerebellar circuits and with the cortex (Diedrichsen et al., 2019). We therefore refrain from making predictions on the direction of the effect. Nonetheless, if cerebellar rTMS is capable of changing performance on social sequence generation, the causal role of the cerebellum for social cognition will be firmly established. An additional benefit of rTMS is that it can be potentially applied for clinical treatment (Klooster et al., 2016).

Taken together, we hypothesized that if the cerebellum is causally involved in the generation of social action sequences, we should observe performance changes on the Picture or Story sequencing task for social action sequences after targeting the cerebellum using LF-rTMS in comparison with a Sham condition.

## Method

### Participants

Participants were 46 right handed, native Dutch speaking individuals (32 females), ages varying from 20 to 36 years (M = 24.64 years; SD = 3.99 years). All participants reported no abnormal neurological history and had normal or corrected-to-normal vision. Informed consent was obtained in a manner approved by the Medical Ethics Committee at the Hospital of University of Ghent, where the study was conducted. Participants were paid 20 euro in exchange for their participation.

### Materials

Participants performed the Picture and Story sequencing task, including practice trials and their respective non-sequential control task as described in Heleven et al. (2019) in which participants saw scenarios consisting of four cartoon-like pictures or sentences that represented (non-social) mechanical events, social scripts, true beliefs, and false beliefs (for an example of a false belief in the Picture sequencing task, see Figure 1). The present experiment was conducted in Dutch, but all versions of this task (in Dutch, French, Italian, and English) are available on request.

### Procedure

Each participant performed the Picture and Story sequencing task. Both tasks were randomly split in two halves that were administered before and after stimulation, in a counterbalanced order across participants. Each task followed the same procedure before and after stimulation. First, participants performed a non-sequential control task where they saw four pictures/sentences in their correct chronological order with a factual question at the bottom of the screen. Participants were instructed to respond to the question as quickly as possible. Second, participants performed the sequencing task. On each trial, they saw four pictures/sentences in a random order, and they had to line up the pictures/sentences in the correct order at a self-paced tempo by indicating the first picture/sentence, then the second, and so on. Each time, the selected picture/sentence moved on the screen along the order indicated.

For the stimulation, we used a double cone coil attached to a Magstim Rapid2 Plus1 magnetic stimulator (Magstim Company Limited, Minneapolis, United States), positioned the coil holder with the center of the coil placed 1–2 cm below the inion, and delivered rTMS at a frequency of 1 Hz, 2 trains of 500 pulses with an intertrain interval of 0.5 s. Participants were sitting in a comfortable chair during stimulation. Half of the participants (*n* = 23) received active stimulation at 80% of their resting motor threshold of their feet. The other half received sham stimulation at 10% of the maximum machine output. All participants wore earplugs and were blindfolded.

### Analysis

For each task and pre- and post-stimulation separately, we calculated the mean accuracy and log transformed response times for correct responses (RTs; see Table 1). We analyzed these data using a repeated measures ANOVA with Sequence Type (mechanical vs. social script vs. true belief vs. false belief vs. non-sequential control) and Time (pre- vs. post-stimulation) as within-participant factors, and Group (TMS vs. Sham) as between-participant factor. To support the hypothesis, a significant Time × Group interaction should show a differential pre-post Time effect for the two Groups. A priori statistical power analyses using GPower3 (Faul et al., 2007) in order to obtain a power of 80% (α = 0.05) with a rather small effect size according to Cohen (1988), revealed that we should include 34 individuals in this study. Given that mainly the posterior cerebellum is targeted by TMS, a preferential effect on social belief sequences would be revealed by an additional interaction with Sequence Type. Two-sided paired samples t-tests were computed to further explore the differences that were significant in the ANOVA, corrected for multiple comparisons using Benjamini Hochberg corrections (Benjamini and Hochberg, 1995) with a false discovery rate (FDR) of 5%.

**TABLE 1:** Mean Accuracy and log transformed Reaction Times of correct trials (in seconds) for the Picture and Story sequencing tasks pre- and post- TMS and Sham.

Picture task – Accuracy										
	
	False belief		True belief		Social script		Mechanical		Control	
**Pre-TMS**	5.63	(0.79)	5.26	(1.01)	5.28	(1.23)	5.91	(0.42)	5.22	(1.35)
**Post-TMS**	5.85	(0.55)	5.35	(1.22)	5.80	(0.52)	5.70	(0.75)	5.48	(1.16)
**Pre-Sham**	5.63	(0.79)	5.22	(1.48)	5.72	(0.72)	5.46	(1.00)	5.48	(1.16)
**Post-Sham**	5.85	(0.51)	5.35	(1.20)	5.91	(0.29)	5.76	(0.71)	5.61	(1.03)

**Story task – Accuracy**										
	
	**False belief**		**True belief**		**Social script**		**Mechanical**		**Control**	

**Pre-TMS**	5.51	(0.56)	5.86	(0.32)	5.82	(0.31)	5.70	(0.54)	5.95	(0.25)
**Post-TMS**	5.77	(0.46)	5.81	(0.34)	6.00	(0.00)	5.74	(0.34)	5.88	(0.39)
**Pre-Sham**	5.27	(0.82)	5.82	(0.36)	5.84	(0.29)	5.72	(0.42)	5.73	(0.53)
**Post-Sham**	5.64	(0.44)	5.83	(0.33)	5.85	(0.29)	5.84	(0.38)	5.88	(0.39)

**Picture task – Reaction times**										
	
	**False belief**		**True belief**		**Social script**		**Mechanical**		**Control**	
	***		***		***		***		***	

**Pre-TMS**	9.99	(0.34)	9.89	(0.27)	9.75	(0.31)	9.83	(0.32)	9.01	(0.42)
**Post-TMS**	9.90	(0.31)	9.87	(0.28)	9.59	(0.20)	9.66	(0.28)	8.88	(0.34)
	—		**—**		**—**		**—**		**—**	
**Pre-Sham**	9.87	(0.24)	10.02	(0.25)	9.80	(0.21)	9.88	(0.23)	9.17	(0.33)
**Post-Sham**	10.03	(0.29)	9.93	(0.31)	9.70	(0.25)	9.77	(0.21)	9.10	(0.37)

**Story task – Reaction times**										
	
	**False belief**		**True belief**		**Social script**		**Mechanical**		**Control**	

	***		***		***		***		***	
**Pre-TMS**	10.20	(0.22)	10.27	(0.30)	9.94	(0.26)	10.00	(0.28)	9.42	(0.30)
**Post-TMS**	10.08	(0.29)	10.11	(0.26)	9.87	(0.28)	9.92	(0.28)	9.22	(0.39)
	*		**—**		**—**		*		*	
**Pre-Sham**	10.32	(0.26)	10.41	(0.28)	10.01	(0.25)	10.17	(0.22)	9.57	(0.23)
**Post-Sham**	10.20	(0.21)	10.21	(0.20)	9.90	(0.17)	9.99	(0.22)	9.37	(0.31)

*Accuracy totals 6 points for each trial and is determined by 2 points for a correct first or last position, and 1 point for the intermediate positions (identical as in Langdon and Coltheart, 1999, and Heleven et al., 2019).*

Reaction times only for trials with accuracy = 6. Numbers between parentheses are standard deviations. Asterisks indicate significant differences from pre to post stimulation for logged reaction times using a two-sided *t*-test with FDR adjusted *p*-values.

*p ≤ 0.015–0.005; ***p < 0.001; —*p >* adjusted threshold.

## Results

Accuracy rates for the Picture and Story Sequencing tests were at ceiling, showing no relevant effects. Of more interest, an ANOVA on RTs showed between group differences between the TMS and Sham group on the Picture sequencing task were significant [*F*(1,44) = 4.65, *p* = 0.036, ηp^2^ = 0.096] and only trending for the Story sequencing task [*F*(1,44) = 3.61, *p* = 0.064, ηp^2^ = 0.076]. No other simple or interaction effects were significant. Mean accuracies and RT’s are listed in Table 1.

**FIGURE 2:**
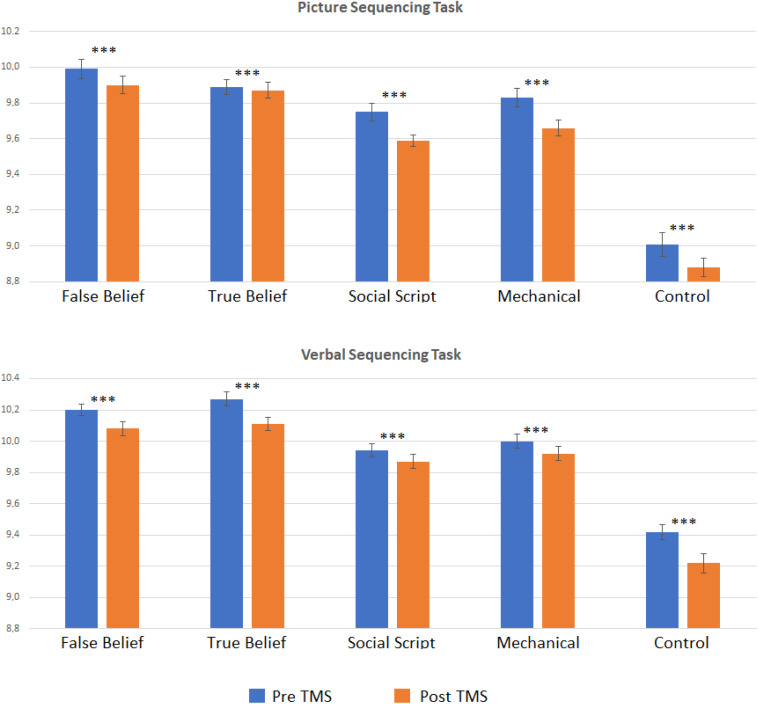
Logged reaction times pre and post TMS per story type (false belief, true belief, social script, mechanical, and non-sequential control) for the Picture and Verbal Sequencing task. Asterisks indicate significant differences from pre to post stimulation for logged reaction times using a two-sided *t*-test with ^∗∗∗^*p* < 0.001 (FDR adjusted). In the sham conditions, these pre to post differences were almost all non-significant, see Table 1.

In order to explore whether the effect was significantly weaker in the Sham than the TMS group, we computed pre-post difference scores per Sequence Type and compared the two groups directly using *t*-tests. However, no significant differences emerged. To further explore our hypotheses, we conducted separate pre- versus post analyses per group. Two-sided paired *t*-tests revealed that the TMS group showed a strong learning effect on both Picture and Story sequencing and for all Sequence Types as reflected in significant decreased RTs from pre- to post-stimulation (adjusted *p* < 0.001). In contrast, the Sham group showed much weaker or no significant learning effects on Story sequencing (adjusted *p* ≤ 0.005–0.015), and no significant learning effects for Picture sequencing. This implies that although the learning effects did not differ significantly between groups, the TMS group showed systematic learning effects clearly above threshold (see Figure 2), while the sham group was below threshold, at least for picture sequencing. Significant levels per Sequence Type are listed in Table 1.

## Discussion

The current preliminary study is a first step to investigate the causal impact of the cerebellum on social sequence generation. Specifically, we investigated the effect of LF-rTMS on performance on the Picture and Story sequencing task in which participants had to generate the correct chronological order of social and non-social actions presented in cartoons or short sentences. Our results revealed a learning effect, reflected in faster response times, from pre to post stimulation on both sequencing tasks. Moreover, for both tasks, we observed these learning effects for all types of sequences (i.e., mechanical, social script, true belief, and false belief). In contrast, participants receiving Sham stimulation showed much weaker learning effects for the Story sequencing task and no learning effects at all for the Picture sequencing task. This suggests that cerebellar LF-rTMS seems to have a positive effect on sequence generation across Sequence Types, and that the cerebellum might be causally involved in the generation of adequate sequences of stories presented in pictures or words. However, the differential learning effects between TMS and Sham groups did not reach statistical significance in an interaction effect when compared directly. Unexpectedly we observed similar effects for our non-sequential control as for the sequential conditions. When participants merely viewed or read different story elements in the correct order, they processed a correct sequence which is inherent to each story. We speculate that our stimulation also influenced this inherent sequence processing in the non-sequential control task. Future studies should further investigate specific effect for sequences, sequence types and modalities.

Since we only measured behavioral data, we have no information on the invoked neural effects of our LF-rTMS. Note that there are many inhibitory projections within cerebellar circuits and with the cortex (Diedrichsen et al., 2019), as was also revealed in the Picture Sequencing task by negative functional connectivity from the cerebellum to the social cortex that can be interpreted as error signals (Van Overwalle et al., 2020). Therefore, the assumed inhibitory effect of LF-rTMS is consistent with cerebellar facilitation in learning as observed in the decrease of reaction times, and this might reflect inhibition of error signals from the cerebellum to the social cortex. Future studies investigating the effects of (LF-r)TMS targeting the cerebellum could use brain stimulation in combination with pre and post measures under the fMRI scanner. This way, specific modulatory effects on neural excitability and connectivity can be revealed (Fox et al., 2012). This will increase our insight on the neural effects in the brain after TMS stimulation.

Although promising, this study had some limitations. First, our choice of a double coil might have weakened potential treatment effects. A double cone coil was chosen for its capacity for deeper stimulation, since our target region was the posterior cerebellum. Unfortunately, this type of coil has wider electric field distributions (Deng et al., 2013). Therefore, it is likely that we stimulated an extensive part of the bilateral cerebellum, including cerebellar areas that supported social as well as non-social sequencing, and this may explain the observed non-differential effects for social and non-social sequences. In addition, this coil cannot always be brought in close proximity of the cerebral skull, as the fit of this device with the head might differ between individuals. A different coil, such as perhaps the traditional figure-of-eight coil, might therefore be more effective as it enables researchers to target a more specific cerebellar region, and might ensure that it is positioned close to the skull for all participants. Moreover, in order to optimize the stimulation of a target region for each participant individually, researchers could turn to fMRI-guided stimulation (Sparing et al., 2008). Second, the current study might have suffered from a lack of power due to a low number of data points. We tested only a relatively small number of persons per group (i.e., 23) and a limited amount of stimulus material available from previous studies (i.e., 4 and 9 stimuli per Sequence Type for the Picture and Story Sequencing task, respectively). Future studies should use a more extensive set of sequencing stimuli so that the present preliminary findings can be confirmed more robustly. Furthermore, this will allow research to use more complex and within-participant designs and might lead to more variation in accuracy rates, enabling researchers to analyze and interpret accuracy findings which was difficult in the present study due to the observed ceiling effect. Lastly, although only 10% of the maximum machine output was applied in the sham, well below the minimum motor threshold of 30%, we cannot completely exclude that this resulted in some kind of neuronal modulation in the sham condition. Future researchers might also consider adding an active control stimulation session. If well-chosen, this active stimulation can also serve as a control for experienced discomfort associated with cerebellar stimulation (Fernandez et al., 2018). However, in the current experiment, since participants’ discomfort immediately ceased when the stimulation stopped, we did not expect effects on task performance after the stimulation.

To conclude, the results of the current study are promising but preliminary, pointing to non-specific effects of LF-rTMS on the cerebellum encompassing various social and non-social sequence types, for both the picture and verbal versions of the task (although some differences were revealed between both tasks). Nonetheless, they seem to suggest a beneficial role for cerebellar LF-rTMS on sequencing. If future studies address the above mentioned limitations, we believe that they might lead to a better understanding of the role of the cerebellum in social cognition, and to potential novel clinical treatment methods for persons suffering from social cerebellar deficits. Moreover, it is possible that not so much a single or stand-alone LF-rTMS treatment, but rather a combination with a behavioral social sequencing training, might lead to the most optimal clinical outcome.

## Data Availability Statement

The raw data supporting the conclusion of this article will be made available by the authors, without undue reservation.

## Ethics Statement

The studies involving human participants were reviewed and approved by Medical Ethics Committee at the Ghent University Hospital. The patients/participants provided their written informed consent to participate in this study.

## Author Contributions

All authors designed the study and protocol together. EH and KD conducted the study, assisted by SD. EH performed the data-analyses and wrote the manuscript.

## Conflict of Interest

The authors declare that the research was conducted in the absence of any commercial or financial relationships that could be construed as a potential conflict of interest.
